# Phytochemical Modulators of Mitochondria: The Search for Chemopreventive Agents and Supportive Therapeutics

**DOI:** 10.3390/ph7090913

**Published:** 2014-09-04

**Authors:** Maja M. Grabacka, Malgorzata Gawin, Malgorzata Pierzchalska

**Affiliations:** Department of Food Biotechnology, Faculty of Food Technology, University of Agriculture, ul. Balicka 122, 30-149 Krakow, Poland

**Keywords:** resveratrol, curcumin, sulforaphane, respiration, retrograde and anterograde signaling, longevity, cancer

## Abstract

Mitochondria are crucially important for maintaining not only the energy homeostasis, but the proper cellular functions in a general sense. Impairment of mitochondrial functions is observed in a broad variety of pathological states such as neoplastic transformations and cancer, neurodegenerative diseases, metabolic disorders and chronic inflammation. Currently, in parallel to the classical drug design approaches, there is an increasing interest in the screening for natural bioactive substances, mainly phytochemicals, in order to develop new therapeutic solutions for the mentioned pathologies. Dietary phytochemicals such as resveratrol, curcumin and sulforaphane are very well tolerated and can effectively complement classical pharmacological therapeutic regimens. In this paper we disscuss the effect of the chosen phytochemicals (e.g., resveratrol, curcumin, sulforaphane) on various aspects of mitochondrial biology, namely mitochondrial biogenesis, membrane potential and reactive oxygen species production, signaling to and from the nucleus and unfolded protein response.

## 1. Introduction

Mitochondria are very specialized organelles that are not only indispensable for maintaining energy homeostasis in eukaryotic cells, but also play prominent roles in cell physiology and fate, including the distinction between survival and apoptotic pathways. The current view on the evolution of life assumes that the presence of mitochondria seems to be an intrinsic property of the last common eukaryotic ancestor [1]. The acquisition of respiratory competent endosymbionts and development of mitochondria as organelles were fundamental for the whole further evolution of eukaryotes and subsequently multicellular organisms. Nevertheless, microsporidians (*Eucarya*, *Protozoa*, *Microsporidia*) serve as an example that not all eukaryotes fully utilize the benefits of respiration or the diverse metabolic pathways offered by mitochondria. In these microorganisms the adaptation to parasitic life has led to structural and functional reduction of cellular components, manifested by the loss of mitochondria. Several taxa retained only mitochondrial relics or mitochondrion-like organelles (MLOs, mitosomes and hydrogenosomes). Interestingly, protists such as the parasitic *Giardia lamblia*, *Trichomonas vaginalis*, *Encephalitozoon cuniculi* or *Cryptosporidium parvum* that had been believed to never possess mitochondria, were later shown to harbor numerous genes of mitochondrial origin in their nuclear genomes [2]. Remarkably, the genes of particular importance were those involved in the iron sulfur (Fe-S) cluster assembly and the Fe-S cluster transfer into apo-acceptor proteins. Three distinct molecular systems responsible for Fe-S cluster biogenesis and maturation of the Fe-S holoproteins have been discovered in Prokaryota, namely ISC (for *iron-sulfur cluster*), SUF (for *sulfur mobilization*) and NIF (for *nitrogen fixation*) [3]. ISC system is common for bacteria and eukaryotic mitochondria. The central role in this system is played by pyridoxal phosphate-dependent cysteine desulfurase, which is a sulfur donor for the cluster. The genes IscU and Isc1 from bacteria and eukaryotes, respectively, encode for small scaffold proteins that create the molecular environment for Fe-S assembly, and they belong to the most evolutionary conservative proteins ever described [3]. Ready-made Fe-S clusters are transferred from the scaffold protein to the recipient apoproteins of various kinds, and next active holoproteins are formed.

This group of metalloproteins is diverse and comprises enzymes of crucial importance for numerous metabolic and biosynthetic pathways, such as respiration (complex I, II and III subunits), Krebs cycle (aconitase), ferredoxin, glutamate or xanthine dehydrogenases, just to mention but a few examples. Noteworthily, the Fe-S biogenesis machinery is also present in mitosomes, hydrogenosomes and mitochondrion-like organelles (MLOs). This fact inspired a hypothesis that Fe-S formation is indispensable for life and is the primary reason for the success of endosymbiosis [4].

Nevertheless, in multicellular organisms mitochondria play crucial roles in metabolism and energy generation, regulation of survival/apoptosis and maintaining the proper cellular redox homeostasis. The frequently used model for studying various aspects of mitochondrial functions and genetic autonomy are rho zero (ρ°) cells that are devoid of mtDNA. They are generated by prolonged culture in the presence of ethidium bromide [5]. Such cells cannot carry on normal electron transport in the respiratory chain due to the lack of critical subunits of complex I, III, IV and F1F0 ATP synthase [6]. Their energetic needs are met exclusively by glycolysis and they are auxotrophic in terms of uridine and pyruvate requirement for growth. Although various metabolic pathways are impaired, these cells are resistant to apoptosis in response to stimuli that induce this type of cell death in normal cells [6]. This is an illustrative example of the role of mitochondria in cell physiology. 

## 2. Role of Mitochondria in the Etiology and Onset of Human Diseases

Mitochondrial dysfunctions underlie many severe pathologies, such as malignant transformation and cancer development, as well as neurodegenerative disorders and type 2 diabetes [7,8]. The pathologies associated with mitochondria can either result from the inherited or acquired mutations within the mitochondrial genome or the cause may lie in the mutated nuclear genes that encode proteins involved in respiration or the mtDNA maintenance. Due to the large number of mtDNA copies in each cell (up to several thousands), the onset of syndromes evoked by mutations in mtDNA depends on homo- or heteroplasmy, and the proportion of mutated mitochondrial genomes in the latter case.

The first group of diseases show diverse severity, affect numerous organs and tissues and manifest by neurological (ataxia, dystonia), ophtalmological (diplopia, ptosis, ophthlmoplegia), gastrointestinal (dysphagia), respiratory (apnoea) and haematological (anemia, pancytopenia) symptoms. Well recognized and described syndromes, such as Leber Hereditary Optic Neuropathy (LHON) or mitochondrial myopathy [9] are relatively easy to diagnose, nevertheless, overall clinical picture can be blurred by the existence of common diseases, such cardiomyopathy or diabetes. There is also the possibility that certain mtDNA variations predispose to diabetes, Alzheimer disease or Parkinson disease [10].

The second group comprises pathologies that originate from mutations in the nuclear genes involved in replication of the mitochondrial genome (such as polymerase γ), mitochondrial transcription (mitochondrial transcription factor A, TFAM) or translation (mitochondrial elongation factor Tu, TUFM). Experiments performed on mice have demonstrated that homozygous mutations in the proof-reading polymerase γ subunit A (PolgA) that lead to accelerated accumulation of subsequent mutations and deletions in mtDNA, cause dramatic decrease in lifespan, premature aging with all the characteristic symptoms (alopecia, spine kyphosis, osteoporosis), as well as loss of subcutaneous fat and reduced fertility [11]. Single nucleotide polymorphisms (SNPs) in TFAM have been recently associated with moderately increased risk of Alzheimer or possibly Parkinson disease development [12,13,14,15,16]. Similarily, certain SNPs discovered in TUFM gene are associated with increased susceptibility to asthma and obesity in North European populations [17].

Oarticular interest has been attracted to the phenomenon of accumulation of mtDNA mutations in cancer. The ground-breaking study by the Vogelstein group showed that mitochondrial genomes in normal cells are generally homoplasmic or exhibit fairly low levels of heteroplasmy that is either inherited from the mother or come from somatic mutations turning up in early embryonic life [18]. Conversely, as demonstrated on colorectal tumor tissue, cancer cells acquire a higher degree of heteroplasmy and the authors noticed that “90% of cancers harbored at least one point mutation not present in the matched normal mucosa” [18]. However, still it is not entirely clear if these mutations actively contribute to the tumor progression or are just merely the result of accumulation of mistakes after hundreds of replications [18,19]. Nevertheless, these mutations are frequently responsible for the widely observed impairment of mitochondrial functions in cancer cells.

In conclusion, mitochondria are regarded as an important target for therapeutic intervention. It is possible to change the “mitochondrial factor” in the onset of many diseases not only by applying some specific drugs, but also by modulating many environmental factors, for example by increasing physical activity or changing dietary habits. The last latter seems particularly important as many current studies indicate that some bioactive food ingredients have a great potential to target mitochondria.

## 3. Dietary Phytochemicals Acting on Mitochondria

Apart from the classical high throughput drug design and synthetic drug development approaches, an extensive search for natural bioactive compounds is being undertaken. Special interest has been focused on phytochemicals, food-derived bioactive compounds and nutraceuticals that might link high biological activity with good tolerance and low systemic toxicity. So far, the efforts have led to the characterization of numerous substances that modulate mitochondrial functions and show promising chemopreventive and anticancer or neuroprotective properties.

Various bioactive phytochemicals present in food were traditionally believed to exert health-promoting effects and currently the number of scientific reports investigating their action in cell culture and animal models has been growing rapidly. Green tea plyphenols (catechins, epigallocatechins, and their derivatives), quercetin, genistein from soybean and allicin from garlic are among the most extensively studied compounds.

Green tea polyphenols, particularly epigallocatechin-3-gallate (EGCG) have been shown to act as potent chemopreventive and anticancer agents. Their mode of action concentrates on induction of mitochondrial apoptotic pathway, e.g., mitochondria depolymerization, cytochrome c release and activation of caspases, in various cancer cell lines, including prostate cancer [20], nasopharyngeal carcinoma [21], melanoma [22], breast cancer [23], pancreatic cancer [24,25], gastric cancer [26], cervical cancer [27,28] and hepatoma cells [29]. Some reports point out that the ROS–related effects may contribute to the antiproliferative and proapoptotic activity of EGCG [29,30,31,32].

Another flavonoid with potential chemopreventive and neuroprotective properties is quercetin, broadly distributed in many plants and vegetables of the human diet. Quercetin was found not only to prevent the reactions to oxidative stress [33] but also actively mount up in mitochondria in biologically active form in cells, during treatment with micromolar concentrations (10–50 μM) [34]. Although the role of this accumulation is not clear, it points to the importance of future studies on mitochondria-polyphenol interactions. Such a study was performed with genistein, an isoflavone that structurally resembles quercetin. Pham and coauthors developed methods of genistein intracellular delivery using nanoemulsions and lipid micelles that showed improved selectivity towards mitochondria. New formulations containing genistein were more cytotoxic for hepatoma and colon carcinoma cells, largely through destabilization of mitochondrial membrane and induction of cytochrome c release leading to apoptosis [35].

Allicin, the most abundant bioactive compound of garlic is well known from its antibacterial activities since the 1940s [36]. Later, other interesting and beneficial effects of allicin have been described, such as antifungal activity [37], high reactivity and anticancer properties [38,39,40]. Allicin is a highly reactive product of alliinase that is formed from the aminoacid alliin. The enzyme (alliinase) and its substrate (alliin) reside in separate intracellular compartments, but after the disruption of the garlic clover tissues they both are released and the reaction takes place [40]. The ability to penetrate biological membranes and high reactivity towards thiol groups make allicin a unique compound with interesting anticancer properties that can be attributed to the alterations it causes in the intracellular redox potential [41]. Allicin has a very short half-life in biological systems and disappears from blood within several minutes [40]. After absorption, allicin rapidly reacts with reduced glutathione (GSH) to form S-allylmercaptoglutathione, which still maintains its strong antioxidant properties [42], but is also more stable and can penetrate tissues and can reach remote organs [40]. The mechanism of allicin’s anticancer action involves the decrease of mitochondrial and cytosolic GSH levels, mitochondrial damage, formation of permeability transition pores (mPTP) that lead to cytochrome c release from mitochondria and subsequent apoptotic pathway.

**Figure 1:**
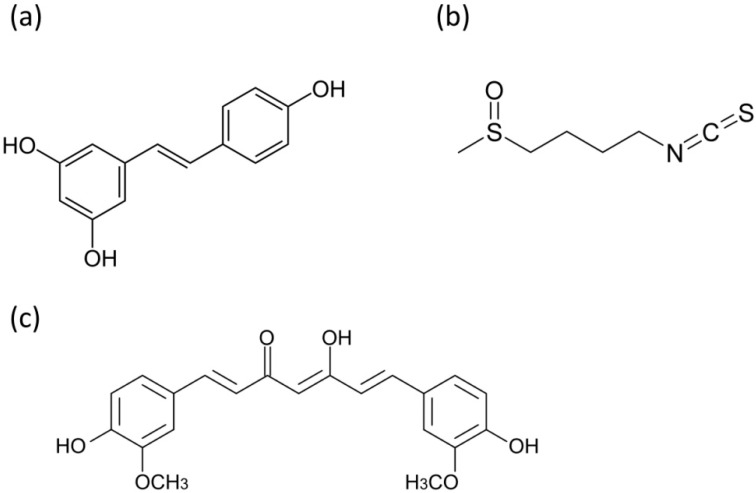
Chemical structures of bioactive phytochemicals (**a**) resveratrol (trans form); (**b**) sulforaphane; (**c**) curcumin.

This review will concentrate on the activities of a dual phenolic compound, the stilbene resveratrol, the ferulic acid derivative curcumin and the isothiocyanate sulforaphane. Resveratrol is a phytoalexin present in fruits, synthesized in plants in response to environmental stress; curcumin is a main ingredient of turmeric or powdered *Curcuma longa* rhizome, and sulforaphane is released from glucosynolates of cruciferous vegetables by myrosinase. The chemical structures of the mentioned compounds are shown in the Figure 1. These three bioactive substances are chemically distinctive and exert specific biological effects, but their common feature is the capability to alter mitochondrial functions and processes. This review will describe four main groups of these phenomena: (1) mitochondrial respiratory function and ATP supply, (2) reactive oxygen species generation and antioxidant protection, (3) mutual communication between mitochondria and nucleus, retro- and anterograde signaling, (4) unfolded protein response and lifespan control. The last part characterizes briefly the impact of phytochemicals on mitochondria in cancer stem cells.

## 4. Mitochondrial Respiration and Energy Generation

The efficiency of ATP generation in mitochondria depends on the proper function of the respiratory chain, maintaining the electrochemical gradient and potential across the inner mitochondrial membrane. Apart from the state of individual mitochondria, global ATP production for the whole cell depends on the number of mitochondria and balance between mitochondrial biogenesis and recycling processes (such as mitophagy). Resveratrol is able to affect all these aspects of mitochondrial biology.

**Figure 2:**
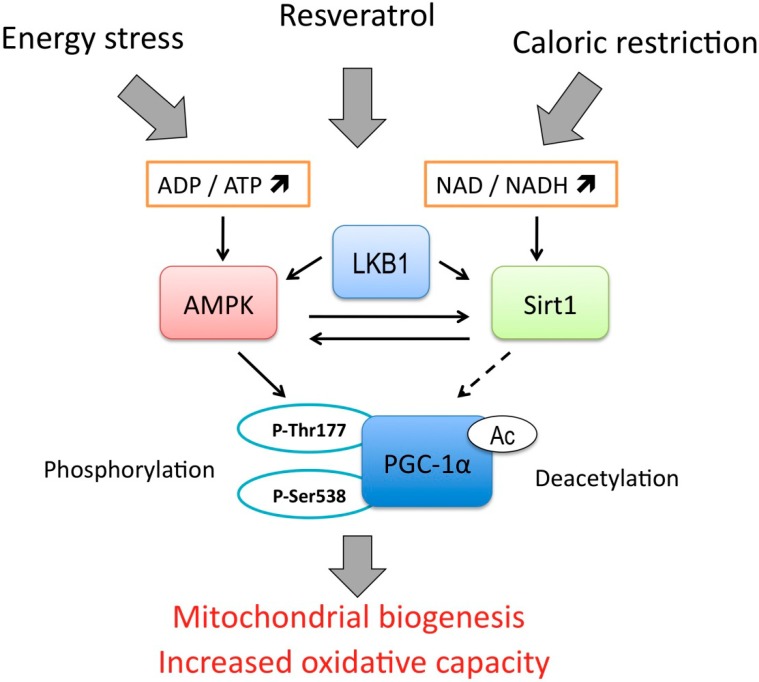
Energy regulated signaling pathway. AMP-dependent kinase (AMPK) and sirtuin 1 (Sirt1) act as energy stress sensors and detect low ATP and NADH levels. Tumor suppressor and upstream kinase LKB1 activates both AMPK and Sirt1. The effector proteins, such as PGC-1α act as transcriptional coactivators and enhance mitochondrial respiratory capacity and biogenesis. Solid black arrows represent activation or up-regulation, dashed black arrow indicates a controversial role of Sirt1 in modulation of PGC-1α activity.

The experiments performed on human breast cancer cell lines cultured *in vitro* and implanted as xenografts in nude mice, revealed a strong antiproliferative effect of resveratrol treatment and induction of apoptosis [43]. The mechanism of apoptosis induction involved the rapid depolarization of mitochondria and release of Ca^2+^ from the endoplasmic reticulum. The disrupted calcium homeostasis together with mitochondrial stress lead to activation of calpain and opening of mPTP, release of cytochrome c and activation of classical caspase dependent pathway [43]. Of note, the loss of mitochondrial membrane potential was induced by relatively high concentration of resveratrol (100 μM). In lower ranges, closer to physiologically achievable concentrations, resveratrol was shown to positively influence mitochondrial performance in mice skeletal and C2C12 myotubes [44]. The concentrations higher than 50 μM were toxic for C2C12 cells, but the “training” with repeated exposure of these cells to 20 μM resveratrol for 6 h interchanged with 18 h recovery periods evoked AMP-dependent protein kinase (AMPK) activation, subsequent PGC-1α (peroxisome proliferator activated recpetor gamma coactivator 1α) activating phosphorylation and increased mitochondrial biogenesis. Both AMPK and PGC-1α are involved in the adaptation to energetic stress and intensified physical activity. Therefore, it seems that resveratrol induces a mild stress in the muscles that serves as a stimulus for increasing the oxidative capacity that translates to improved running endurance *in vivo* tests with mice [44]. Interestingly, the reported effects of resveratrol were completely independent on protein deacetylase sirtuin 1 (Sirt1). Sirt1 had previously been regarded as the main protein target of resveratrol, although the later studies showed that it is not able to activate Sirt1 directly [45,46]. Sirt1 is activated during caloric restriction and nutrient deprivation and acts along with AMPK with mutual activating interplay between these two proteins (the signaling pathway is shown in the Figure 2). PGC-1α acts as the main transcription regulator governing the mitochondrial biogenesis, and is activated both by AMPK-driven phosphorylation on Ser 538 and Thr 177 [47] and deacetylated by Sirt1 [48,49]. The latest results, however, revealed that in fact deacetylation of PGC-1α by overexpressed Sirt1 decreased its activity as a transcriptional coactivator and prevented resveratrol induced mitochondrial biogenesis [44].

The enhancement of mitochondrial oxidative capacity in muscles is a hallmark of exercise-induced improvement in physical performance. Numerous studies performed on rodents show the beneficial effects of dietary supplementation with resveratrol on the mitochondrial activity (measured as oxygen consumption and activity of respiratory and lipid-oxidizing mitochondrial enzymes) and biogenesis (measured as the increase in mtDNA content and protein levels of mitochondrial transcription factor A and PGC-1α) [50,51,52].

AMPK activation is necessary for resveratrol induced mitochondrial biogenesis, whereas Sirt1 is not [52,53]. Interestingly, the effects of resveratrol on the exercise performance depend also on the overall physical fitness. The experiments with rats selectively bred for high or low running performance demonstrated that resveratrol enhanced training response in the former but not the latter animals [51,54]. Resveratrol exerts also beneficial effects in mice fed on high fat diet, particularly in decreasing body weight, improving glucose response and reducing ROS levels in muscles [52]. Significant increase in the uncoupling proteins UCP1, UCP2 and UCP3 in white and brown adipose tissue of mice treated with resveratrol suggested that the body weight loss could be attributed to enhanced energy expenditure due uncoupling of substrate oxidation from ATP synthesis [52]. These effects seem to be a promising strategy against metabolic syndrome and type 2 diabetes that are currently severe health concern in developed societies.

Similarly to resveratrol, curcumin has been reported to act as a mitochondrial uncoupler through its protonophoric activity [55]. As a protonophore, curcumin induced the state 4 respiration (with glutamate and malate provided as substrates for complex I, not producing ATP) of isolated rat liver mitochondria. Additionally, in a dose dependent manner it activated F0F1-ATPase, which is a common feature for protonophoric uncouplers.

In many cancerous cell lines sulforaphane, the main isothiocyanate of cruciferous vegetables, inhibits cell proliferation and induces apoptosis when applied in physiological concentrations (even as low as 5 μM) and these events were shown to be connected with disruption of mitochondrial membrane potential. However, the same compound in similar range of concentrations was shown to preserve mitochondrial functions in many stress situations (ischemia or toxin-induced damages) especially in normal noncancerous cells [56]. These apparently contradictory experimental observations must be reconciled on the ground of the molecular mechanism of action of sulforaphane in cells.

**Figure 3:**
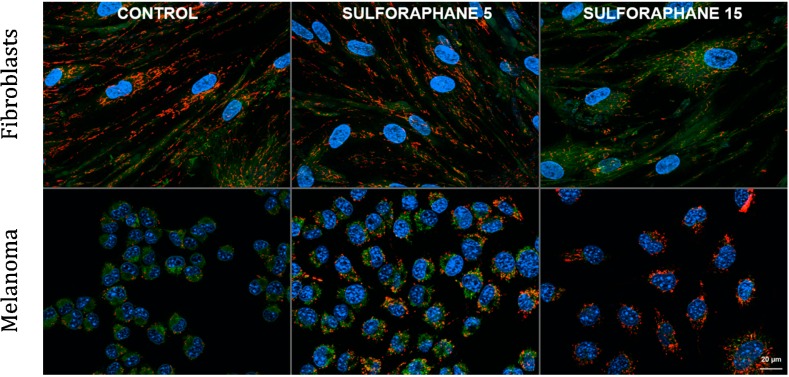
Suforaphane differentially modulates mitochondrial membrane potential in normal and transformed cells. Control (vehicle treated) and sulforaphane (5 and 15 μM, 24 h incubation) treated cells were stained with JC-1 potential sensitive dye; polarized mitochondria are stained red, mitochondria with compromised potential/depolarized are stained green. The nuclei are counterstained with Hoechst 33342. Upper row: nontransformed human fibroblasts, bottom row: B16 F10 mouse melanoma cells.

Our own experiments demonstrated the interesting discrepancy in the action of sulforaphane on normal human fibroblasts and malignant melanoma B16 F10 cells (Figure 3, unpublished data). In fibroblasts, nontoxic concentrations of sulforaphane (5 and 15 μM) seem to slightly decrease the mitochondrial membrane potential in a dose-dependent way. In contrast, B16 F10 cells respond to the same doses of sulforaphane with the increase of mitochondrial membrane polarization (Figure 3). The possible explanation could be that isothiocyanate activates the mechanism of restoration of the mitochondrial potential, generally lower in the malignant cells, to the level characteristic for nontransformed cells. Therefore sulforaphane action is manifested by the higher percentage of polarized mitochondria. On the contrary, normal cells would adjust the intensity of mitochondrial activity to the current energy demands, perhaps lower in the sulforaphane treated cells with slowed down proliferation rate than in control.

Currently the pleiotropic influence of sulforaphane on many aspects of cell physiology is attributed to two main mechanisms. First, sulforaphane, called an indirect antioxidant, is a strong activator of Keap1/Nrf2/ARE pathway responsible for the induction of ROS-dissipating and detoxifying enzymes. Secondly, well-documented targets of sulforaphane are histon deacetylases (HDAC). As a potent inhibitor of HDAC expression and activity it causes epigenetic modifications of many genes involved in proliferation, DNA repair and apoptosis [57].

NFE2L2 (Nuclear factor (erythroid-derived 2)-like 2, also known as Nrf2) was recently shown to regulate not only stress related genes but also respiration. NFE2L2 is constitutively upregulated in many cancerous cells unresponsive to chemotherapy and marking the poor prognosis for patients [58]. The cells with NFE2L2 genetic knockout have lower level of mitochondrial potential and impaired ATP production by oxidative phosphorylation. In agreement, knockout of Keap1, leading to constant NFE2L2 activation, causes an increase in mitochondrial potential and cellular ATP levels. All these differences are shown to be independent of respiratory chain impairment but associated with the changes in the supply of reduced NADH and FADH_2_ [59]. Consequently, sulforaphane as NFE2L2 inducer should beneficially influence mitochondria. What is more, it was shown in the experiments performed on human fibroblasts (both normal and isolated from muscle dystrophic patients) that sulforaphane induces mitochondrial biogenesis [60].

## 5. Reactive Oxygen Species and Oxidative Stress

The antioxidative activity of resveratrol is undoubted and has been confirmed by a great number of reports. It was demonstrated that in various cell lines, malignant and non-transformed, primary cultures and freshly isolated tissues, resveratrol decreased total ROS level and generation of superoxide anions in mitochondria in particular. Moreover, resveratrol restores the proper level of antioxidant protection by replenishing glutathione levels and inducing expression of ROS scavenging enzymes, such as MnSOD and inhibits lipid peroxidation triggered by metal-induced radicals [61,62].

In this context, the finding that increased mitochondrial capacity stimulated by physical exercise was actually ROS dependent, and indeed ROS generated in muscles during exhaustive activity were the stimulus that triggered mitochondrial biogenesis was quite unexpected [63]. Besides respiratory chain activity, the major source of ROS during exhaustive exercise is xanthine oxidase. Administration of antioxidative supplements, such as vitamin C or allopurinol (xanthine oxidase inhibitor) results in significantly weaker post-training performance in rats, comparing to control groups [63]. These data indicate that the enhancement of exercise performance, mentioned in the section above, in response to resveratrol could not be attributed to its antioxidative properties.

Cytoprotective effects associated with the antioxidative activity of resveratrol have been observed in renal epithelium, cardiomyocytes, neurons and brain tissue, retinal pigment epithelium and other cells [64,65,66,67,68]. For example, neuroprotective action of resveratrol was described in the brains of rats subjected to hypoxia/reoxygenation induced injury, and additionally it significantly accelerated the regeneration of mitochondria after the administration of synthetic uncoupler, CCCP [69]. Interesting neuroprotective effects are exerted by resveratrol in traumatic brain injury, as well as LPS challenge and involve anti-inflammatory activity towards microglia, manifested by downregulation of IL-1, IL-6, IL-12, TNFα and nitric oxide release [70,71,72].

These cytoprotective effects, however, are not observed in cancer cells, which respond to resveratrol with impairment of mitochondrial function, disruption of calcium homeostasis that leads to calcium-induced calcium release (CICR) from mitochondria; opening of membrane permeability transition pore and apoptosis in consequence [73,74]. These opposite effects observed in malignant *vs.* nontransformed cells suggest that there are distinct physiological features that differentiate the response to the same molecule in these two cellular environments. The possible reason of such contrasting outcomes could involve different basal intracellular ROS level (lower in healthy cells with fully functional mitochondria) and the efficiency of antioxidative protection mechanisms, particularly glutathione levels, catalase, superoxide dismutases activities, *etc.* (which are usually lower in transformed cells).

The case of curcumin activity may be similar, since there are numerous reports showing its antioxidative properties, as well as conversely, demonstrating the striking elevation of the generated ROS. For example curcumin was shown to induce mitochondrial damage and subsequent apoptosis in lung cancer cells, Jurkat cells or mouse fibroblasts [75,76,77]. Cytoprotective effects of curcumin against oxidative injuries were reported for adipocytes differentiated from mesenchymal stem cells [78] HepG2 cells [79], neonatal rat lung [80], retinal pigment epithelial cells [81], spinal cord astrocytes [82] or hepatocytes [83].

Another possible explanation for the cytoprotective and cytotoxic activities of curcumin in various experimental settings is the variability in the doses used. Chang and collaborators showed that high doses of curcumin (80 μM) increased intracellular ROS levels, induced mitochondrial damage and cytochrome c release and subsequenty apoptosis in osteosarcoma cells, whereas low concentration (10 μM) actually reduced ROS levels and did not show any toxicity [84]. This also raises questions about curcumin biavailability *in vivo* and the range of achievable plasma concentrations of curcumin. The low bioavailability has been regarded as an obstacle in the way of launching various phytopharmaceuticals into clinical therapies.

Bioavailabilty of dietary plant phenolic compounds is quite low and plasma concentrations of flavonoids, catechins, proanthocyanidins vary but never exceed concentrations (C_max_) of 10 μM in plasma, and usually are much lower [85]. Curcumin bioavailability is particularly weak due to low solubility and instability in water and even very high oral doses (12 g daily) result in undetectable plasma concentrations with the detection limit of 1 μg/mL [86]. For resveratrol, oral doses of 250 mg result with 2 μM peak plasma concentration of this polyphenol and its metabolites [87]. The low plasma concentration of polyphenols is not an effect of weak absorption, but rather a very rapid metabolism, glucuronidation and sulfation [87]. In order to enhance bioavailability and improve pharmacokinetic parameters various experimental drug delivery systems have been tested for both curcumin and resveratrol, including laurosyl sulphate or stearyl and oligisaccharide chitosan carrier [88,89], poly-lactic-co-glycolic acid (PLGA) nanoparticles [90,91]. Nevertheless, rapid metabolism of polyphenols in human digestive tract and relatively short cleareance of these substances are the factors that limit their delivery to the specific cellular targets.

With this caveat in mind, chemical modifications were applied to these compounds with the purpose of enhancing their absorption and cell targeting. An example of a successful strategy was the development of new curcumin derivatives with remarkable affinity to mitochondria, which has been recently reported by Reddy and colleagues [92]. Curcumin was conjugated with triphenylphosphonium lipophilic cations to obtain Mitocur-1, -2 and -3 molecules that exhibited significantly enhanced mitochondrial accumulation. These compounds in 10 μM concentrations exerted high toxicity in breast cancer cells but not normal mammary epithelial cells. The anticancer effects involved cell cycle arrest, increased superoxide generation, loss of mitochondrial membrane potential and subsequently apoptosis [92].

Sulforaphane also has a contradictory role in establishing cellular ROS homeostasis. After entering cells and/or mitochondria it is immediately conjugated to GSH what leads to profound GSH depletion and could result in increase ROS generation in the first hours of incubation with the compound. In the longer run however, it enhances the expression of many ROS controlling enzymes, as including NAD(P)H:quinone oxidoreductase-1 (NQO1), heme oxygenase-1 (HO-1), GSTs (gluthatione S-transferases) what makes it an potential antioxidant [93]. The ROS threshold theory states that in cancerous cells, which generally are characterized by increased ROS levels, the intensive ROS production caused by sulphorafane could pass the threshold of death signals. In normal cells the same increase of ROS level will evoke the cytoprotective effect due to lower initial ROS value [56].

## 6. Signaling between Mitochondria and Nucleus

Acquisition of bacterial endosymbionts in eukaryotic ancestor cells enforced the development of bidirectional signaling mechanisms between the mitochondrion and nucleus. Anterograde (nucleus to mitochondria) and retrograde (mitochondria to nucleus) signaling pathways are ancient and early developed communication routes that coordinate the mitochondrial response to changing intracellular microenvironment and act as sensor mechanisms governing the cellular response to external, e.g., nutritional stimuli.

**Figure 4:**
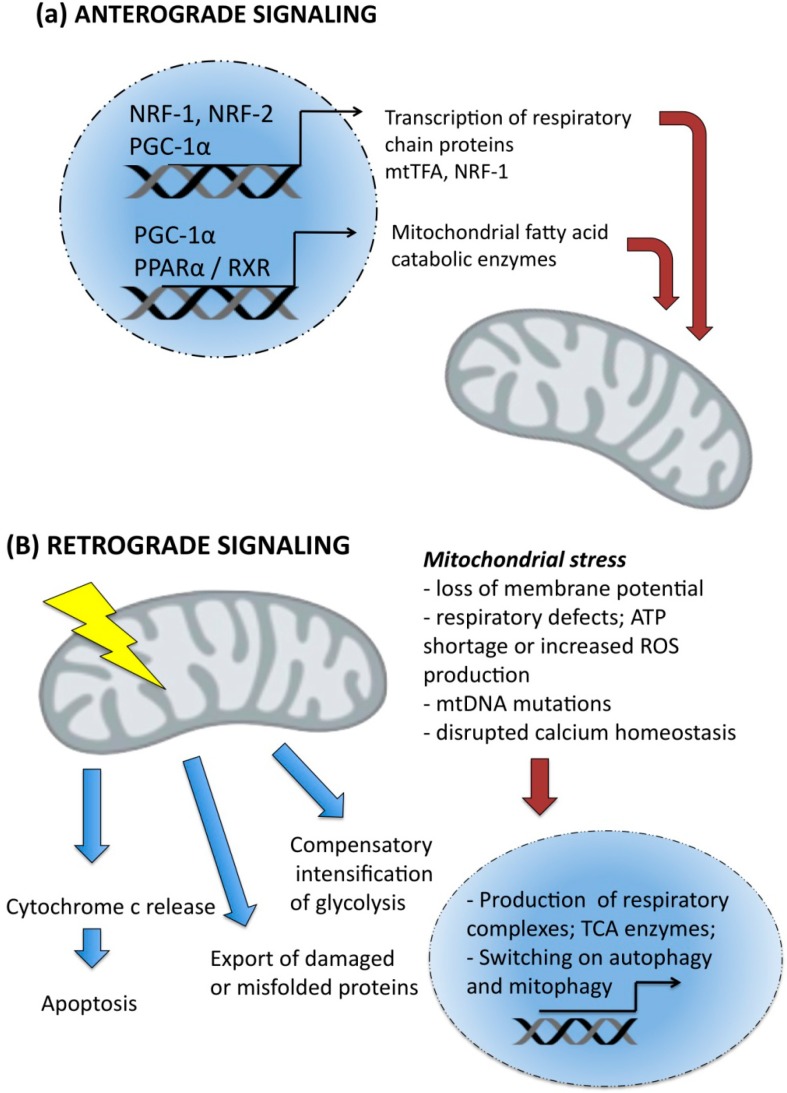
(**a**) Anterograde; (**b**) retrograde signaling pathways. Explanations are given in the text.

Anterograde signaling existence is a result of the gradual loss of mitochondrial autonomy in terms of regulation of transcritpion and translation. Evolution had led to the transfer of increasing numbers of genes encoding the proteins crucial for respiration to nuclear genome. Mitochondrial DNA encodes only 13 subunits of respiratory complexes I, III, IV and V, although they are indispensable for electron transport and respiration. Nuclei have taken over the significant control expression of respiratory complexes, but also numerous proteins involved in the maintenance and replication of mtDNA or enzymatic machinery driving various metabolic pathways in the mitochondrial matrix [94,95]. This control over mitochondrial biogenesis and metabolic activities is held by nuclear respiratory factors, NRF-1 and NRF-2 (coincidentally the same abbreviation is used in the literature for nuclear respiratory factor 2 and for nuclear factor (erythroid derived 2)—like 2 activated by sulforphane and mentioned in Section 3). NRF-1 binding sites were found in numerous genes encoding the respiratory complexes subunits, but also others responsible for their assembly, import and exerting auxiliary functions or involved in heme biosynthesis [96]. NRF-1 is an essential gene, as was shown in mice with disrupted DNA-binding and dimerization domains. Homozygous NRF1 null mice showed early embryonic lethality and even blastocysts were unable to grow in culture [94]. NRF-1 can bind to PGC-1α and together they transactivate respiratory genes, mitochondrial transcription factor A (TFAM), a direct regulator of mitochondrial DNA replication/transcription and NRF-1 itself, acting as a positive regulatory loop, that is fundamentally important for mitochondrial biogenesis [94,97]. PGC-1α acts in cooperation with peroxisome proliferator activated receptor α (PPARα), which is a transcription factor activated upon ligand binding. PGC-1α/PPARα induce transcription of enzymes from fatty acid β-oxidation pathway and uncoupling proteins, UCP1 and possibly other uncoupling proteins, UCP2 and UCP3 that play important role in thermogenesis after exposure to cold [98,99,100,101,102,103]. PGC-1α interacts also with general coactivators such as CBP/p300 and SRC-1 that possess histone acetyltransferase activity. PGC-1α belongs to unique class of coactivators that can simultaneously induce transcription and mRNA processing when bound to the cognate promoter of a target gene. The C-terminal domain of PGC-1α has an RNA recognition motif and a region responsible for recruitment of splicing factors [104]. Two other transcriptional coactivators related to PGC-1α have been identified, namely PGC-1α related coactivator (PRC) and PGC-1β. They cooperate with NRF-1 and induce mitochondrial biogenesis in similar way to PGC-1α [94], but their tissue distribution is different and does not overlap with PGC-1α (Figure 4a).

Retrograde signaling evolved as a cellular adaptation to factors and conditions that impair mitochondrial functions. When mitochondria emit such a signal to the nucleus the cell can switch on repair programs and reorganize metabolism to keep energetic homeostasis [105]. Retrograde signaling is induced by defects in the respiratory chain, accumulation of mtDNA mutations, alterations in mtDNA copy number or loss of membrane potential. This pathway is best described in yeast *Saccharomyces cerevisiae*. A marker gene of retrograde response is peroxisomal citrate synthase CIT2, which is greatly induced by mitochondrial injury in yeast. CIT2 expression is driven by two basic leucine zipper transcription factors Rtg1 and Rtg3, and cytoplasmic regulatory protein Rtg2. Rtg2 requires ATP binding for its activity and is modulated by ammonia, glutamine and glutamate levels, that suggest it acts as a metabolic sensor. In yeast, Rtg proteins play role in integration of metabolism and mitochondria maintenance by: (1) upregulation of peroxisomal glyoxalate cycle for oxidation of fatty acids, (2) sustaining anaplerosis via truncated Krebs cycle that provides intermediates for glutamate and lysine biosynthesis, (3) activating aconitase as a bifunctional Krebs cycle enzyme but also stabilizing mtDNA [106]. In mammalian cells the retrograde response is much more diverse and less well defined, but comprises several main events, such as upregulation of some nuclear encoded mitochondrial proteins (citrate synthase, cytochrome oxidase CoxVa) and non-mitochondrial like cytoskeleton (β-actin), glycolytic (GAPDH) or signaling (c-Myc) proteins. Other symptoms of retrograde response involve imbalanced calcium homeostasis, elevated cytoplasmic Ca^2+^ levels, increased expression of endoplasmic reticulum calcium release channels; low ATP levels and compensatory enhancement of glycolysis [107]. These events trigger stress signaling associated with activation of calcineurin, NFκB, MAPK and PKC. Subsequently, insulin growth factor 1 receptor membrane levels are up-regulated, which enables intensification of glucose uptake (Figure 4b). In some way the cascade of these events resembles the hallmarks of cancer: particular avidity for glucose, high rate of aerobic glycolysis (Warburg effect) and overactive mitogenic signaling. For that reason, Guha and coworkers proposed a hypothesis that mitochondrial damage by induction of retrograde response might be indeed the cause of oncogenic transformation, rather than its result [105]. Resveratrol and curcumin act on both anterograde and retrograde signaling pathways. First, they activate PGC-1α, a canonical regulator of mitochondrial biogenesis and as well as AMPK, which coordinates the metabolism in order to keep balance between anabolic and catabolic processes and preserve cellular energy stores. In this context, resveratrol helps to protect energetic homeostasis. On the other hand, the antioxidative and inflammatory properties of resveratrol reflect in reduced intracellular ROS levels, and inhibition of inflammatory (i.a NFκB) signaling [108,109,110]. These processes decrease the sensitivity of mitochondria to superoxide-induced damage or mitochondrial membrane lipid peroxidation that could trigger the retrograde response. It would be interesting to speculate that the chemopreventive activity of resveratrol could be linked to silencing of retrograde responses.

An alternative mode of action has been presented in a recent report by Jeong and coauthors, who describe the differential effect of a synthetic, more stable resveratrol derivative HS-1793 and pure resveratrol on mitochondria in MCF-7 cells [111]. Although both compounds decreased membrane polarization and ATP levels, HS-1793 significantly down-regulated TFAM and TUFM levels responsible for the mitochondrial transcription and translation that are crucial for mitochondrial maintenance in the cells. Consequently, HS-1793 treated cells were not able to make up for the dwindling ATP stocks with enhanced mitochondrial biogenesis and eventually die [111]. In this context, despite being nuclear genes, TFAM and TUFM can be regarded as attractive molecular targets for phytochemical based therapy that open possibility to block entire mitochondrial transcription and translation and break out the energetic catastrophe in cancer cells.

Sulforaphane was recently shown to enhance the expression of the transcriptional coregulator SPBP in HeLa cells which in turn activates p62/SQSTM1 [112,113]—the protein considered by some researchers to be one of main regulators of autophagy and mitophagy [113]. If this is the case, sulforaphane action on cells would result in keeping the mitochondrial homeostasis by elimination of damaged mitochondria and induction of mitochondrial biogenesis. Similarly, resveratrol and its chemically modified analogue with improved stability, Longevinex^®^, have recently been shown to induce mitophagy in response to ischaemia and reperfusion injury in rat heart [114], which facilitates remodeling of damaged mitochondria and therefore accelerates cellular regeneration.

## 7. Unfolded Protein Response and Longevity

Unfolded protein response is a mode of retrograde signaling which is turned on when damaged mitochondria release peptides from their matrix into the cytoplasm [115]. Short peptides generated in mitochondria from misfolded proteins can activate certain nuclear transcription factors that belong to the retrograde pathway.

**Figure 5:**
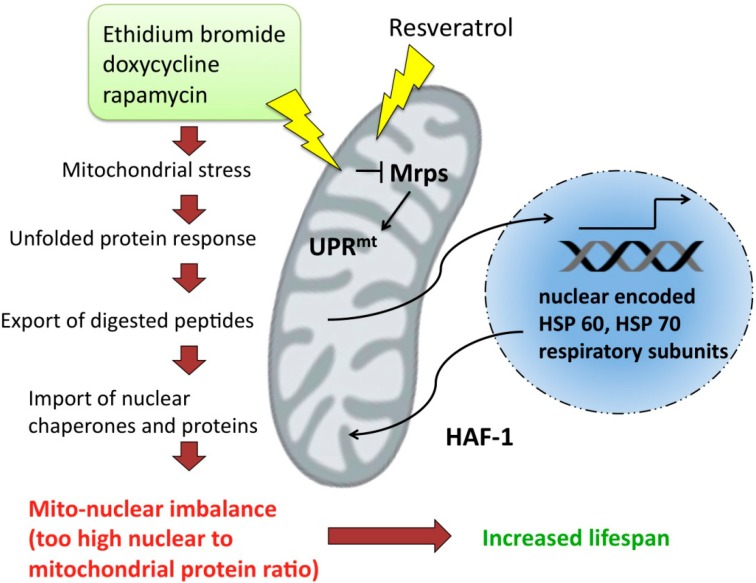
Mitochondrial stress induced by antibiotics, as well as resveratrol, leads to unfolded protein response and subsequently to the increase in nuclear encoded protein import to mitochondria.

Thirteen proteins encoded in mtDNA have to cooperate with nuclear encoded proteins to sustain the proper functions of mitochondria. The mitochondrial translation machinery, including mitochondrial ribosomal proteins (Mrps) is necessary not only for biosynthesis of these 13 proteins, but also for maintaining a particular stoichiometric ratio between nuclear and mitochondrial respiratory proteins. Alteration of this ratio creates a stimulus that triggers mitochondrial unfolded protein respose (UPR^mt^). The fact, that some antibacterial antibiotics, as well as ethidium bromide, specifically inhibit transcription, is fundamental to experimental approaches developed to study unfolded protein response. In the interesting study by Houtkooper *et al*. [116], the authors show that unfolded protein response determines longevity in various eukaryotic organisms. The study revealed that Mrps5 expression level decreases with age and moreover, the knockdown of this gene during developmental stage extends the lifespan of *Caenorhabditis elegans* [116]. Interestingly, Mrps5 knockdown led to increased motility of worms, reduced basal respiration, and reduced ATP and citrate synthase activity. Silencing of Mrps5 by RNAi altered mitochondrial translation and induced mitonuclear imbalance (a stoichiometric imbalance between nDNA and mtDNA encoded respiratory proteins). Reduced levels of Mrps5 induce mitochondrial stress and subsequently activate UPR^mt^ and import of HSP-60 and HSP-70 chaperones that are meant to restore a prober protein condition. Hiperactive UPR^mt^ correlates with significant extension of lifespan. UPR^mt^ involves upregulation of a mitochondrial peptide transporter HAF-1 and ubiquitin-like protein 5 (UBL5) that induces transcription of mitochondrial chaperones. Other factors that induce mitonuclear imbalance, such as antibiotic inhibitors of mitochondrial transcription (doxycycline or rapamycin) exert similar effects to knock down of Mrps5 and increase lifespan in worms and mice [117,118]. Interestingly, resveratrol shows similar activity and increases the proportion of nDNA encoded OXPHOS subunits over mtDNA encoded ones. This effect does not lead to the drop in ATP levels or in basal respiration intensity, but conversely, resveratrol increases oxidative phosphorylation, maintains ATP levels and citrate synthase activity unaltered (Figure 5). This indicates that resveratrol does not induce energetic stress.

Even though proper mitochondrial activity and efficient respiration are hallmarks of cellular health, it has been reported that the reduction of mitochondrial activity can increase longevity. The studies performed on yeast, *C. elegans*, *Drosophila* and mice indicate that inactivating mutations in respiratory complex components and subsequent impairment in mitochondrial activity increase lifespan [119,120,121,122]. The possible explanation stating that reduced mitochondrial activity and oxidative metabolism is associated with reduced ROS generation in the mitochondria, and therefore lower oxidative damage, does not find support in the experimental evidence. Increased oxidative stress does not shorten the lifespan of *C. elegans* [123,124]. RNAi mediated silencing of cytochrome c oxidase-1 subunit Vb (cco-1) in the larval stages of *C. elegans* could induce increased lifespan in adulthood, even when the silencing is limited to certain tissues (intestine or neurons) [125]. Lifespan extension due to cco-1 silencing turns on UPR^mt^, and this type of UPR is necessary for prolongation of life, but not cytoplasmic or endoplasmic reticular UPR [125].

UPR is triggered in mitochondria during the problems to achieve a proper structure of respiratory complexes, including Fe-S cluster assemby and transfer to apoproteins. A newly discovered nutrient-deprivation autophagy factor (NAF-1) belongs to the group of proteins responsible for Fe-S cluster transfer to recipient molecules and localizes both in endoplasmic reticulum and mitochondrial outer membrane [126]. One of its client proteins is ferredoxin [126]. Resveratrol has been shown to interact with NAF-1 and formation of this complex enhances stability of Fe-S cluster bound NAF-1. In result, resveratrol abrogates the cluster transfer from NAF-1 to recipient protein or to mitochondria [126]. Although no direct physiological implications of NAF-1/resveratrol interaction have been revealed, we may speculate that inhibition of the Fe-S cluster transfers leads to the increase of not fully functional apoproteins in the mitochondria which subsequently could induce UPR^mt^ and further exert a longevity-extending pathway.

## 8. Mitochondria in Cancer Stem Cells

Recent views on the origin of cancer highlight the importance of a small subpopulation of cells, called cancer stem cells (CSCs). These cells are present in tumors and being particularly difficult to destroy, are regarded as the main reason of the limited success of existing therapies. But on the other hand, CSCs represent a new, interesting target for prospective cancer treatment.

The intrinsic property of CSCs is the ability of asymmetric division, which means that one of the daughter cells maintains a self-renewal potential and the second gives rise to all cell types of the general cancer population. Therefore, they are also called tumor-initiating cells [127]. The first mention of the CSCs dates to 1963, when Bruce and Van der Gaag observed that a small number of murine lymphoma cells were capable of proliferation *in vivo* [128]. Most convincing evidences for cancer stem cells theory were provided from the studies on acute myeloid leukemia (AML). In 1994 Lapidot and colleagues identified the CD34+ CD38− fractions of AML cells, which were capable to AML initiation after transplantation into severe combined immune-deficient (SCID) mice [129]. Similarly, Bonnet and Dick observed leukemic blasts generation after infusion of the CD34+ CD38− cells into non-obese diabetic, severe combined immune-deficient (NOD/SCID) mice [130]. This rare cell population exhibited potential for self-renewal and the ability to differentiate and proliferate. Thereafter, most studies in CSCs that have been conducted allowed the identification of this kind of cell population in various type of carcinoma, like pancreatic cancer [131,132], brain tumor [133], lung cancer [134], bladder cancer [135], prostate cancer [136], melanoma [137], ovarian cancer [138], neck and head cancer [139], colon cancer [140], colorectal cancer [141], hepatocellular carcinoma [142], liver cancer [143] and breast cancer [144].

The origin of CSCs is still unknown, but several theories were proposed. One theory assumes that accumulation of mutations in normal stem cells or progenitor cells leads to CSC formation [145]. The origin of CSCs from normal stem/progenitor cells is also indicated by some similarities between this type of cells, like expression of molecular markers or cellular phenotype and size [146,147,148]. Another theory suggests that genetic and epigenetic factors could cause de-differentiation or transdifferentiation due to horizontal gene transfer and cell fusion in mature somatic cells [149]. The major obstacle in testing the emerging hypotheses is that currently a universal, unequivocal method for CSC identification does not exist.

However, particular cell culture environments have been shown to facilitate CSC detection. For example, spheroid culture of cancer cells leads to enrichment of spheroids in stem cells-like cells. Spheroids generated from primary ovarian cancer and human ovarian cancer cell lines contain cells which are able to self-renewal, proliferation, differentiation, tumor formation, metastasis and resistance to chemotherapy [150]. Moreover, this method has been used to enrich various CSCs like prostate CSC [151], colon CSC [152], brain CSC [133], breast CSC [153].

There are interesting differences in the mitochondrial structure and function between stem cells and normal mature cells. Mitochondria in stem cells are immature, filamentous, have poorly developed cristae, and are localized in the perinuclear region. Due to the limited functionality of these mitochondria in respect to ATP generation, stem cells’ metabolism is based on glycolysis. When the differentation program is switched on, the mitochondria undergo maturation manifested by the increase in amount of mtDNA, formation of the complex network and cristae and enhanced capability of oxidative phosphorylation [154]. Ye and coworkers [155] demonstrated that mitochondrial features characteristic of pluripotent cells occurred also in CSCs. Mitochondria in lung CSCs exhibit perinuclear arrangement, low amounts of mtDNA, lower concentrations of ATP and ROS, higher mitochondrial membrane potential and reduced oxygen consumption. The authors suggested that this traits and properties can be applied as indicators of stemness in normal stem cells as well as in CSCs.

Non-tumorogenic cancer cells produce large amount of ROS that promote fast proliferation and this situation can be the reason for the sensitivity of these cells to radio- and chemotherapy. On the contrary, CSCs are characterized by lower ROS level, low proliferation rate and overexpression of genes involved in protection against oxidative stress. Thus, CSCs exhibit improved capacity of neutralization of intracellular ROS due to the increased production of free radical scavengers.

In the frame of the seeking efficient methods to target CSCs, several phytochemicals have been tested. Tang *et al.* [156] demonstrated that epigallocathechin gallate (EGCG) inhibited growth of prostate cancer stem cells and this effect can be enhanced by using quercetin. EGCG induced mitochondria-dependent apoptosis through caspase-3/7 activation and inhibition of Bcl-2, XIAP and survivin expression in prostate CSCs. Moreover, EGCG with quercetin were able to inhibition of self-renewal and block CSCs migration and invasion. Likewise, resveratrol is capable of effectively inhibiting pancreatic CSCs characteristic in Kras^G12D^ mice. Like EGCG, resveratrol induced caspase-3/7 activity and inhibited expression of apoptosis related proteins, like Bcl-2 and XIAP in human pancreatic CSCs. Furthermore, resveratrol inhibited expression of Sox-2, Oct-4 and Nanog, which are the key factors involved in maintaining pluripotency [157]. Sulforaphane also restrained the self-renewal ability of pancreatic CSCs, mainly by inhibition of the Bcl-2 and XIAP expression, caspase-3 activation and apoptosis induction. What is more, sulforaphane synergized with quercetin and also eliminated CSC-characteristic [158]. Alvero *et al.* [159] showed that treatment of ovarian CSCs with NV-128 (a phenyl-substituted isoflavone compound) induced cell death through two independent pathways. The first was associated with the increased mitochondrial superoxide and hydrogen peroxide production and the second with declines in ATP, Cox-I and Cox-IV levels. Both routes lead to impairment of mitochondrial function and energetic stress. Importantly, these authors demonstrated that manipulation with mitochondrial bioenergetics could induce cell death in ovarian CSCs, which are resistant to proapoptic chemotherapeutics. Curcumin can also be used in anti-cancer therapy targeted to the CSCs. Fong *et al.* [160] demonstrated that this phytochemical effectively decreases the population of cells with CSC characteristics (the so-called side population) in rat C6 glioma, and therefore represents a potentially better solution for glioma patients routinely treated with temozolomide, which actually can increase the side population fraction within glioma cells [161]. On the basis of these results it can by hypothesized that many beneficial effects of resveratrol, curcumin or sulforaphane on tumors development, growth and metastasis *in vivo* may be explained by the influence of dietary phytochemicals on mitochondria mainly in CSCs, not on mitochondria of the whole population of cancer cells of the treated tumors. We should bear in mind this possibility when planning future *in vitro* experiments, for example by employing 3D spheroids culture of tumor cells which contains more CSCs in comparison to normal 2D monolayers.

## 9. Conclusions and Perspectives

Dietary phytopharmaceuticals have been shown to affect various aspects of mitochondrial biology, including energy generation, bidirectional signaling to and from the nucleus, unfolded protein response, apoptotic pathways and stem cell functioning. The knowledge gathered from *in vivo* and *in vitro* studies has demonstrated encouraging effects potentially alleviating the symptoms of various pathologies, such as cancer, neurodegenerative diseases or metabolic syndrome. Nevertheless, the contradictory data about the mechanisms of sulforaphane, resveratrol and curcumin actions need meticulous clarification.

There are certain specific obstacles that hamper the efforts of researchers who investigate phytochemicals in hope to improve chemoprevention: (1) the most severe is weak bioavailability of the natural compounds; (2) the lack of reliable cell culture and animal models to study dietary agents, (3) numerous difficulties in conducting clinical studies. Overcoming these difficulties is an important challenge for future research. What’s more, most of studied phytochemicals have many active derivatives—of note the biodiversity and abundance of chemical modifications are practically unlimited and even subtle changes in chemical structure can profoundly affect the biological functions [162]. The great majority of *in vitro* and *in vivo* studies, however, are conducted on a few popular phytochemicals. In this context, the influence of many “better” analogs of most studied phytochemicals (created by nature or by chemical modification) on mitochondrial functions should be carefully followed, especially these involving modern “omics” technology that allows simultaneous analysis of many chemicals and cell components. Such data are scarce (a resveratrol analog from blackberries, pterostilbene, which was proven to upregulate genes involved in mitochondrial functions, serves as a good example [163].

Such studies, if carefully planned and conducted will draw attention to important advantages of phytopharmaceuticals as supplementary therapeutic agents *i.e.*, the absence of systemic toxicity, diverse health promoting activities, low costs and broad availability comparing to the modern synthetic pharmacuticals. The important challenge is also to attract health professionals from both developing and highly industrialized countries around the world, who are concerned about affordable public medical care to the subject of phytochemicals use in prevention and treatment of diseases. The good news is that concluding from the numerous scientific reports we should value natural diets and traditional cooking heritage of many cultures that propagate awareness of benefits connected to red wine (containing resveratrol), spices (curcumin), vegetables (garlic containing allicin; broccoli and cabbage containing sulforaphane, quercetin), green tea (epigallocatechin-3-gallate) and soy products (genistein) consumption.
